# 3D Hollow Hierarchical Structures Based on 1D BiOCl Nanorods Intersected with 2D Bi_2_WO_6_ Nanosheets for Efficient Photocatalysis Under Visible Light

**DOI:** 10.3390/nano9030322

**Published:** 2019-03-01

**Authors:** Yongchao Ma, Chao Lv, Jiahui Hou, Shaoteng Yuan, Yanru Wang, Ping Xu, Ge Gao, Jinsheng Shi

**Affiliations:** Department of Chemistry and Pharmaceutical Science, Qingdao Agricultural University, Qingdao 266109, China; yongchaoma@126.com (Y.M.); 18306392155@163.com (C.L.); HouJiaHuiMay@163.com (J.H.); 17852029986@163.com (S.Y.); wangyanru09@163.com (Y.W.); 17863975310@163.com (P.X.); GaoGe160607@163.com (G.G.)

**Keywords:** chemical engineering, hierarchical hollow structure, interfacial structure, carrier extraction, photocatalysis

## Abstract

Constructing elaborate catalysts to prompt the charge carrier separation and transport is critical to developing efficient photocatalytic systems. Here, a hierarchical hollow structure based on 1D/2D BiOCl/Bi_2_WO_6_ hybrid materials was fabricated by a precursor chemical engineering method. This hybrid is made up of molten 1D BiOCl nanorods and 2D Bi_2_WO_6_ nanosheets. The synergetic effect of the presence of BiOCl and specific interfaces between BiOCl and Bi_2_WO_6_ provided efficient interfacial charge transfer of photogenerated carriers under visible light. Seamless BiOCl functions like a noble metal, with platinum-like behavior, accelerating the oxidizing ability of fabricated BiOCl/Bi_2_WO_6_ hybrids, which was favorable for the photocatalytic decomposition of organic compounds (3.2 times greater for Rhodamine B (RhB) and 4 times greater for Ciprofloxacin (CIP)) over the Bi_2_WO_6_ catalysts. The beneficial interfacial interaction between BiOCl and Bi_2_WO_6_ resulting from the unique construction prompted the charge transfer from the conduction band of Bi_2_WO_6_ to that of BiOCl. The findings presented in this study provide a cost-effective precursor-mediated strategy to realize the critical and efficient separation of photoinduced carriers in environmental remediation applications.

## 1. Introduction

Photocatalysis has been regarded as a cost-effective technology to realize the goal of clean energy and environmental sustainability [1,2]. Developing semiconductor-based photocatalysts with high charge carrier transfer and separation can effectively improve the photocatalytic activity [3]. Bi_2_WO_6_ is a layered Aurivillius oxide, consisting of [Bi_2_O_2_]^2+^ layers sandwiched between WO_4_^2−^ slabs [4,5]. The small band gap (about 2.5 eV) enables it to absorb the visible light, and the valence band with deep lying characteristics is conducive to oxidation reactions. These advantages make Bi_2_WO_6_ a promising alternative to effectively decompose organic pollutants. However, the rapid recombination of photogenerated carriers within Bi_2_WO_6_ hinders it practical applications. 

Recently, numerous methodologies have been employed for photoactive materials to enhance the photoactivity, including morphology and facet control [6], surface modification [7] and coupling with other photocatalysts [8]. For Bi_2_WO_6_, constructing a heterojunction with a matched band gap is one of the most efficient routes, such as in g-C_3_N_4_/Bi_2_WO_6_ [9], Bi_2_WO_6_/MXene [10] and Bi_2_WO_6_/RGO [11]. According to the reported studies, BiOCl with a layered structure and a large band gap can serve as a potential candidate for photocatalysis [12,13,14]. The layered structure inhibits the recombination of photogenerated carrier pairs between [Bi_2_O_2_]^2+^ and Cl layers [15]. On the other hand, the larger band gap provides sufficient charge separation between the photoinduced carriers. Previously, we prepared BiOCl@Bi_2_WO_6_ composite microspheres via a two-step method [16]. However, the techniques require complex process controls and the degradation performance is not desirable. Therefore, we seek to develop cost-effective strategies for the production of the combined favorable effects of visible light-responsive Bi_2_WO_6_, layer-structured BiOCl, and the efficient separation of photogenerated carriers resulting from heterojunction to advance photocatalytic efficiency.

In the studies described here, an in situ one-pot hydrothermal approach was developed to prepare BiOCl/Bi_2_WO_6_ hybrids via a precursor chemical engineering strategy. The hybrids were characteristic of a hollow hierarchical structure, consisting of 2D Bi_2_WO_6_ nanosheets grown on the surface of 1D BiOCl nanorods. More importantly, the fabricated hybrid materials exhibit a remarkable photocatalytic decomposition of organic compounds utilizing visible light. The wavelength distribution of Xe lamp that was used in photocatalytic reactions was shown in Figure S1. The desirable photoactivity of the developed BiOCl/Bi_2_WO_6_ hybrids is ascribed to the combined effect of favorable band structures and the efficient separation of photogenerated carrier pairs.

## 2. Materials and Methods 

### 2.1. Synthesis of BiOCl/Bi_2_WO_6_ Heterogeneous Hybrids 

The reagents and materials used were of analytic grade. Typically, we first prepared the transparent solution by mixing Bi(NO_3_)_3_·5H_2_O (3 mmol) and ethylene glycol (EG, 60 mL) through stirring. Then, 2 mmol of KCl was added and dissolved into the above solution. After that, 0.5 mmol of Na_2_WO_4_·2H_2_O was added slowly into the above solution. The resultant mixture was poured into a Teflon-lined autoclave (100 mL) at 180 °C for 12 h. The resulting solid was washed repeatedly with ethanol and water and then dried at 80 °C for 6 h. 

For comparison, a series of xCl-yW composites were prepared, labeled as xCl-yW (i.e., 1.5Cl-0.75W, 1Cl-1W and 0.5Cl-1.25W), respectively. The characters x and y represent the molar amount of KCl and Na_2_WO_4_·2H_2_O used in the reaction system, respectively. In addition, pristine BiOCl and Bi_2_WO_6_ were obtained under the same experimental conditions in the absence of Na_2_WO_4_·2H_2_O and KCl, respectively.

### 2.2. Characterization

The shape and detailed microstructure of the prepared product were characterized by field-emission scanning electron microscopy (FE-SEM; S-4800, Hitachi, Ltd., Tokyo, Japan) operated at an acceleration voltage of 5.0 kV, and the corresponding crystalline structure was analyzed using an X-ray diffractometer (XRD; D/MAX-RB, Rigaku Co., Tokyo, Japan) with Cu Kα radiation (λ) 1.5418 Å. Transmission electron microscopy (TEM) and high-resolution transmission electron microscopy (HRTEM) observations were carried out on a JEM-2100F (JEOL Ltd., Tokyo, Japan). UV-vis absorption spectra were studied and recorded on a UV-Vis spectrometer (UV-2550, Shimadzu Co., Kyoto, Japan) equipped with an integrating sphere. The N_2_ adsorption–desorption isotherm and Barrett–Joyner–Halenda (BJH) pore size distribution were analyzed on a Micromeritics ASAP 2020 nitrogen adsorption instrument (Micromeritics Instrument Co., Norcross, GR, USA). The photoluminescence (PL) spectra were investigated at room temperature with a Hitachi (Hitachi, Ltd., Tokyo, Japan) F-4600 fluorescence spectrometer. The electron spin resonance (ESR) spectra of the obtained samples were recorded on an ESR spectrometer (JEOL JES-FA 200, JEOL Ltd., Tokyo, Japan). 

## 3. Results and Discussion

**Figure 1:**
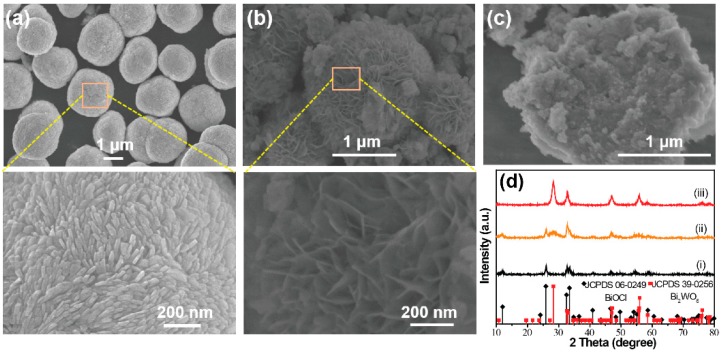
Field-emission scanning electron microscopy (FESEM) images of the as-prepared samples: (**a**) BiOCl, (**b**) BiOCl/Bi_2_WO_6_ hybrids, (**c**) Bi_2_WO_6_. (**d**) The related X-ray diffractometer (XRD) patterns (i) BiOCl, (ii) BiOCl/Bi_2_WO_6_ hybrids and (iii) Bi_2_WO_6_.

The shape and microstructure of the prepared samples were examined by SEM. As indicated in Figure 1, the BiOCl was uniform and made up of monodisperse microspheres, which had an average diameter of about 2 µm. The magnified SEM image shows that the BiOCl microsphere consisted of nanorods. The XRD pattern (Figure 1d) of the as-synthesized BiOCl microspheres indicated that a pure phase of BiOCl was obtained (JCPDS No. 06-0249). When the WO_4_^2−^ ions were mixed with Cl^−^ ions during the preparation, the FESEM image of the prepared hybrid materials showed a flower-like morphology as a whole. The primary unit of the hybrid microspheres were packed ‘petals’, which can be clearly observed in the enlarged part of the SEM image in Figure 1b. The diffraction pattern (ii) in Figure 1d presents the XRD patterns of the hybrid materials. It was found that the hybrid materials were mainly indexed to the tetragonal BiOCl (JCPDS No. 06-0249). Besides, another peak at around 28° can be easily observed, corresponding to the orthorhombic Bi_2_WO_6_ (JCPDS No. 39-0256) [17]. As shown in Figure 1c, the SEM image of pure Bi_2_WO_6_ showed a plate-like shape assembled from nanosheets. The corresponding XRD pattern in Figure 1f confirmed the formation of the pure orthorhombic Bi_2_WO_6_ phase.

We also investigated the effect of competition reaction of Cl^−^ and WO_4_^2−^ ions on the morphology and phase structure of Bi-based hybrid materials in this system, as shown in Figure S2. When the amount of WO_4_^2−^ ions used was 0.75 mmol (Figure S2a), the morphology of the composite was a mixture of particles and spheres. However, the surface texture of the microspheres became rougher. When the amount of WO_4_^2−^ ions used was higher than 0.75 mmol, the microspherical morphological feature of the hybrids was not observed (Figure S2b,c). These results indicate that the precursor chemical engineering strategy was a precondition for the construction of the 3D hierarchical morphology. The phase component of the as-prepared samples was studied by XRD. As shown in Figure S2d, the XRD patterns of the Bi-based hybrid materials confirmed the co-existence of BiOCl and Bi_2_WO_6_. In detail, the diffraction peaks belonging to the Bi_2_WO_6_ phase were gradually intensified with the increased amount of WO_4_^2−^ ions in the reaction system.

**Figure 2:**
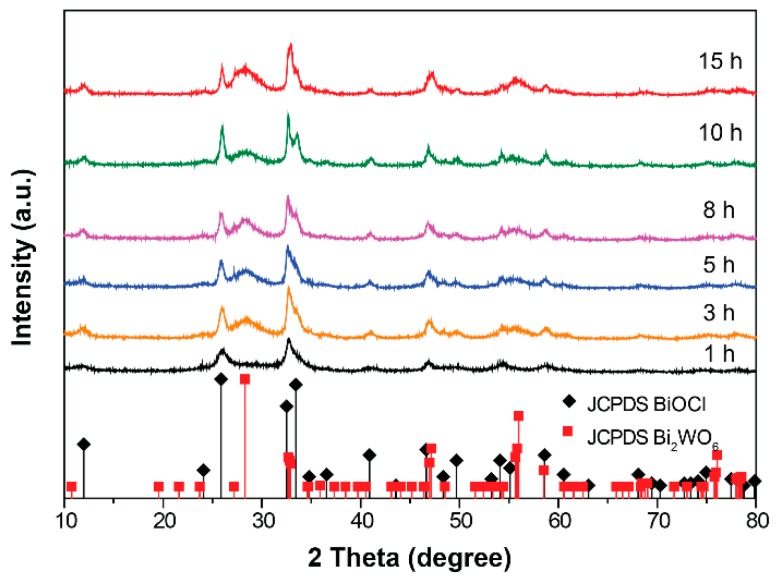
The evolution of XRD patterns of prepared samples as a function of hydrothermal reaction time.

Furthermore, the time-dependent phase structure evolution of the hybrid catalysts was investigated, as shown in Figure 2. During the early hydrothermal stage (t = 1 h), only the diffraction patterns of the BiOCl phase could be observed, which may be due to the low formation energy of BiOCl compared to Bi_2_WO_6_ [18]. However, from 3 h on, we found the appearance and gradual enhancement of the diffraction intensity assigned to the Bi_2_WO_6_ phase. Based on this evidence, it can be concluded that during the growth process of hybrids, BiOCl first forms and then serves as the nucleation site, benefitting the nucleation and growth of Bi_2_WO_6_. A schematic flow chart for the synthesis of BiOCl/Bi_2_WO_6_ hybrid materials is shown in Figure S3.

**Figure 3:**
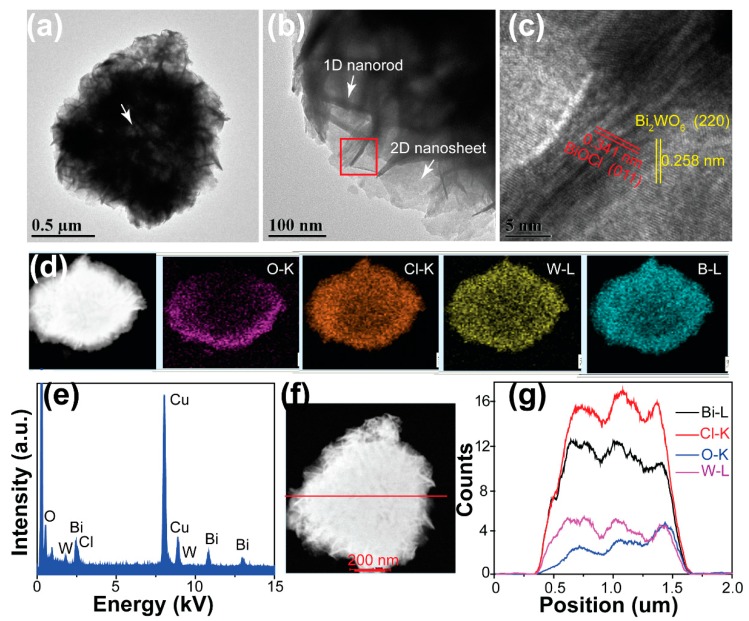
(**a**) Low-magnification and (**b**) high-magnification transmission electron microscopy (TEM) images, (**c**) high-resolution transmission electron microscopy (HRTEM), (**d**) High-angle annular dark field scanning transmission electron microscopy (HAADF-STEM) image and mapping results, and (**e**) EDX spectra of the BiOCl/Bi_2_WO_6_ hybrids. (**g**) Line scanning profiles of Bi, Cl, O and W recorded along the line shown in (**f**).

We then investigated the interfacial interaction between BiOCl and Bi_2_WO_6_ in the BiOCl/ Bi_2_WO_6_ hybrids. Figure 3a,b presents the TEM images of the obtained BiOCl/Bi_2_WO_6_ hybrids, implying the successful combination of the 1D BiOCl nanorods and the 2D Bi_2_WO_6_ nanosheets, as well as the hollow characteristic of the hybrid (white arrow in Figure 3a). The HRTEM image was further studied, as shown in Figure 3c. The sample exhibited two orientations with different spacing distances (about 0.258 and 0.341 nm). These distances were assigned to the crystal spacing of (220) and (011) of Bi_2_WO_6_ and BiOCl, respectively. Combined with the SEM analyses, we concluded that the heterogeneous hybrids consisted of nanorod sheets stacked with BiOCl and Bi_2_WO_6_, which was further confirmed by TEM to be of another kind of 1.5Cl-0.75W composite, as shown in Figure S4. It was observed that the 1.5Cl-0.75W composites were composed of nanorods and nanosheets. In addition, it can be concluded that the Bi_2_WO_6_ nanosheets were grown on the backbone of the BiOBr nanorods because of the favorable formation of BiOCl phase in the reaction system, which is consistent with the XRD analysis in Figure 2. The interface between Bi_2_WO_6_ and BiOCl was clearly observed (Figure S4c). On the other hand, from the scanning transmission electron microscopy (STEM) images of the hybrids (Figure 3d), we observed that the distribution of Cl and O elements was homogeneous in the characterized sample. This finding indicates that the BiOCl and Bi_2_WO_6_ were integrated seamlessly, which was further confirmed by the line scanning profiles (Figure 3f,g). We also observed the denser distribution of all elements along the edges of the hybrids than in the center. In addition, the corresponding energy dispersive spectroscopy (EDS) mapping provides evidence of the presence of Bi, O, Cl and W elements Figure 3e. All these findings indicate that we fabricated a hybrid with excellent interface interaction.

**Figure 4:**
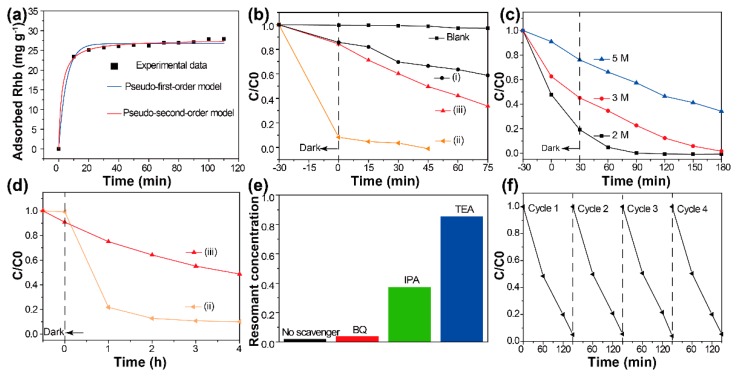
(**a**) Kinetics of Rhodamine B (RhB) adsorption onto the BiOCl/Bi_2_WO_6_ hybrids. (**b**) Removal of RhB solution (1 M) with time over the as-prepared samples: (i) BiOCl, (ii) BiOCl/Bi_2_WO_6_ hybrids and (iii) Bi_2_WO_6_. (**c**) The photocatalytic activity of the RhB solution with different concentrations over the BiOCl/ Bi_2_WO_6_ hybrids: 2 M, 3 M and 5 M, respectively. (**d**) The photocatalytic degradation of ciprofloxacin (CIP) solution by (ii) BiOCl/Bi_2_WO_6_ hybrids and (iii) Bi_2_WO_6_. (**e**) Reactive species trapping experiments and (**f**) Stability experiment of BiOCl/Bi_2_WO_6_ hybrids: RhB solution (2 M).

It has been revealed that photocatalytic reactions always occur at the surface of catalysts [19,20]. The target should be attached to the surface of photocatalysts. To investigate the adsorption behaviors of the developed hybrid materials, dark adsorption experiments were carried out (details in the supporting information). Figure 4a shows the change in adsorbed RhB as a function of contact time. Clearly, the initial adsorption of RhB is very rapid. After 30 min of contact with BiOCl/Bi_2_WO_6_ hybrids, equilibrium adsorption capacity was almost achieved. The adsorption kinetics of RhB molecules was calculated using the Lagergren-first-order and pseudo-second-order models, and the results indicate that the adsorption process was chemisorption [21,22]. The related parameters are summarized in Table S1.

Then, the visible-light activities of the obtained samples were first investigated for the degradation of the RhB solution (details in the supporting information). Prior to irradiation, adsorption equilibrium was achieved. The concentration changes of the RhB solution are shown in Figure 4b. The decomposition degree of BiOCl and Bi_2_WO_6_ for RhB were 45% and 70%, respectively. It has been demonstrated that the degradation of RhB from BiOCl was due to the self-sensitized process [23]. In addition, the BiOCl/Bi_2_WO_6_ hybrids displayed significantly efficient removal rates when compared with pristine BiOCl and Bi_2_WO_6_. It can be speculated that the photocatalysts adsorbed most of the RhB from the solution during the 30 min adsorption period and converted the remainder during visible light exposure. 

To confirm that the decolorization of RhB in aqueous solution really resulted from the photocatalysis, we performed a series of photocatalytic tests with different concentrations of RhB solution, as shown in Figure 4c. It can be clearly seen that the adsorption of the composites decreased gradually with increasing concentrations of RhB solution. A series of corresponding UV-vis spectra are shown in Figure S5. In the adsorption stage, strong adsorption was only observed in the presence of photocatalysts. Under the visible light, a hypochromic shift of the peak located at 553 nm was observed. This result can be ascribed to the demethylation of RhB [24]. Besides, we also confirm the superior photocatalytic degradation of our prepared BiOCl/Bi_2_WO_6_ hybrids towards the ciprofloxacin (CIP) solution, which was four times higher than that of the pure Bi_2_WO_6_ sample (Figure 4d). Compared with our previous report [16], both RhB and CIP decomposed more efficiently under the visible light with the BiOCl/Bi_2_WO_6_ hybrids. 

The decomposition of organic compounds under visible light is mainly driven by the active free radicals and holes produced by the photocatalysts [25]. Thus, we performed related trapping experiments to detect the main oxidative species. As shown in Figure 4e, the photocatalytic activity of BiOCl/Bi_2_WO_6_ hybrids was largely hindered by the triethanolamine (TEA) and isopropyl alcohol (IPA). As a result, the holes and OH were regarded as the dominant active species. Stability is also a critical factor for the practical application of photocatalysts [26]. After four cycles, no obvious change was observed, indicating the good photocatalytic stability of the BiOCl/Bi_2_WO_6_ hybrids (Figure 4f).

**Figure 5:**
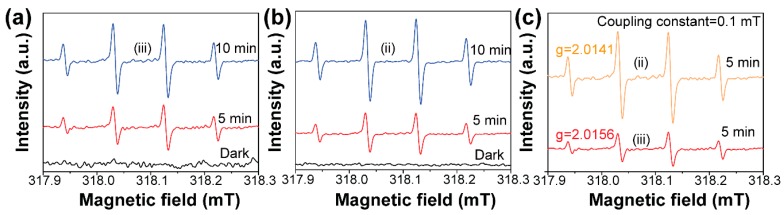
DMPO spin-trapping electron spin resonance (ESR) spectra recorded for ·OH under visible light with various illumination time for (**a**) Bi_2_WO_6_ and (**b**) BiOCl/ Bi_2_WO_6_ hybrids. (**c**) The intensity comparison of production of BiOCl/ Bi_2_WO_6_ (ii) and Bi_2_WO_6_ (iii) under 5 min illumination.

We used the electron spin resonance technology to qualify the formation difference of ·OH species under visible light [27,28]. The results are shown in Figure 5. It was found that the strong ESR spectra with relative intensities of 1:2:2:1 corresponding to DMPO-·OH adduct were clearly observed for the Bi_2_WO_6_ (Figure 5a) and BiOCl/Bi_2_WO_6_ hybrids (Figure 5b). Under the dark condition, no DMPO-·OH signals were detected for the Bi_2_WO_6_ and BiOCl/Bi_2_WO_6_ hybrid materials. This result suggested that the precondition for formation of the ·OH species is the presence of visible light. In addition, as shown in Figure 5c, the DMPO-·OH signal intensity from the BiOCl/Bi_2_WO_6_ hybrid materials was much higher with respect to Bi_2_WO_6_. Thus, the presence of BiOCl was beneficial for the more effective separating ability of photogenerated carriers because of the favorable heterojunction effect.

**Figure 6:**
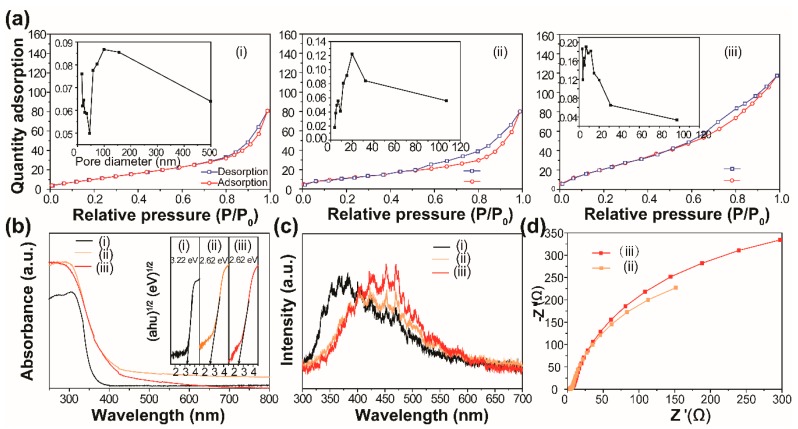
(**a**) Brunner−Emmet−Teller (BET) surface area analysis, (**b**) UV-vis diffuse reflectance spectra and (**c**) Photoluminescence (PL) of the as-prepared samples: (i) BiOCl, (ii) BiOCl/Bi_2_WO_6_ and (iii) Bi_2_WO_6_. Insets (a) and (b) are the corresponding pore size distribution and band gap energies of the prepared samples, respectively. (**d**) The electrochemical impedance spectra (EIS) spectra of (ii) BiOCl/Bi_2_WO_6_ and (iii) Bi_2_WO_6_.

Finally, we explored the reasons for the excellent photocatalytic ability of our developed materials. The N_2_ adsorption–desorption analyses were first performed, and the results are shown in Figure 6a. The summary of the BET surface area, average pore diameter and pore volume of the prepared samples is shown in Table S2. Compared with pure BiOCl, the pore size distribution of the Bi_2_WO_6_ and BiOCl/Bi_2_WO_6_ hybrids showed a mesoporous structure [29,30]. The pore distribution of BiOCl/Bi_2_WO_6_ hybrids was in the range of 2–50 nm, which is helpful for absorbing RhB molecules. 

The light responsive abilities of the BiOCl, Bi_2_WO_6_ and BiOCl/Bi_2_WO_6_ hybrid materials were studied, and the results are shown in Figure 6b. The strong enhancement of the absorption at about 450 nm for the hybrid materials was due to the band–band transition of Bi_2_WO_6_ [31,32]. Also shown is that an absorption edge of BiOCl was located at 380 nm. The spectrum of the BiOCl/Bi_2_WO_6_ hybrids showed the combination of the two spectra of BiOCl and Bi_2_WO_6_. In addition, the absorption intensity of the BiOCl/Bi_2_WO_6_ hybrids was higher in the visible light region compared with pure BiOCl and Bi_2_WO_6_. As a result, the hybrid materials produced more photogenerated charge carriers. The band gap energy (Eg) of these samples can be obtained by the following formula [33,34]: αhν = A (hν–Eg)^n/2^. In this equation, α, ν, and Eg represent the absorption coefficient, light frequency and band gap, respectively. For BiOCl and Bi_2_WO_6_, the values of n were both 4, as for an indirect transition. Therefore, the Eg of BiOCl and Bi_2_WO_6_ was estimated to 3.22 and 2.61 eV from a plot, as shown in the inset of Figure 6b. 

Furthermore, to quantify the transfer efficiency of photogenerated charge carriers, the Photoluminescence (PL) spectra of the samples were performed, and the results are shown in Figure 6c. It was demonstrated that the decreased PL emission intensity provided evidence of the decreased recombination rate of photogenerated carriers. Clearly, in the case of the curve shape, the PL spectrum of the BiOCl/Bi_2_WO_6_ hybrid materials remained the PL feature of Bi_2_WO_6_. However, for the emission intensity, it was found that after combination with BiOCl, the emission intensity of Bi_2_WO_6_ decreased, indicating that BiOCl can serve as an acceptor for the transferred electron from Bi_2_WO_6_ and finally slow down the direct recombination of electrons and holes [35]. On the other hand, the transport dynamics of the interfacial charge were also studied by electrochemical impedance spectra (EIS) (details in the supporting information), as shown in Figure 6d. It can be seen that the electrode based on the BiOCl/Bi_2_WO_6_ hybrid materials had a depressed semicircle in the high frequency range. Thus, we concluded that there was low interfacial resistance between the current collector and active material, low electrolyte resistance, as well as low charge transfer resistance [32]. After the fitting, the values of charge transfer resistance (Rct) for sample ii and sample iii were 710 and 883 Ω, respectively. As shown in Figure S6, the higher photocurrent of BiOCl/Bi_2_WO_6_ indicated more efficient charge separation and better collection efficiency [13].

**Figure 7:**
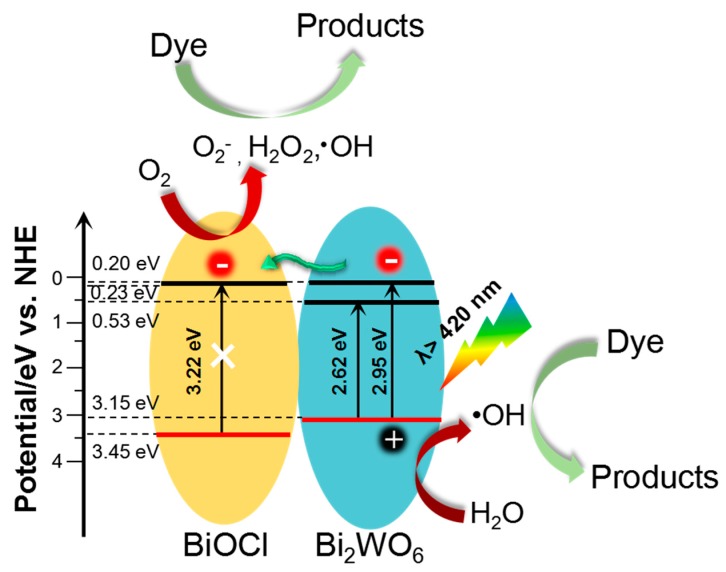
Schematic diagram of the charge–transfer mechanism of BiOCl/Bi_2_WO_6_ hybrids under visible light irradiation.

Based on the above analyses, as depicted in Figure 7, we provided explanations for the enhanced photocatalytic activity of the BiOCl/Bi_2_WO_6_. On the one hand, like other typical BiOCl/ Bi_2_WO_6_ composites, the band edge positions of our developed hybrid materials were obtained from the electronegativity concept [36], as shown in Table S3. Under visible light (Eg ≤ 2.95 eV), Bi_2_WO_6_ with a narrow band gap energy (Eg = 2.61 eV) can be photoexcited to produce electron–hole pairs. In detail, the electrons in the valence band of Bi_2_WO_6_ are excited and then jump to a higher potential edge (0.2 eV). [37,38] The produced electrons are then transported to the conduction band of BiOCl, accelerating the separation of the electron–hole pairs generated by Bi_2_WO_6_. This transference effectively hinders the charge combination, resulting in enhanced photocatalytic activity. On the other hand, the unique structure of our constructed hybrid materials is beneficial for the abundant contact of photocatalysts with organic compounds, and the decreased transport distance of photogenerated carriers resulting from the favorable dimensions of the primary unit (i.e., 2D Bi_2_WO_6_ nanosheets and1D BiOCl nanorods).

## 4. Conclusions

In summary, 3D Bi_2_WO_6_/BiOCl hybrids with hollow and hierarchical characteristics were successfully synthesized in situ via a facile chemical engineering method. The morphology and structure characterization results showed that the 2D Bi_2_WO_6_ nanosheets were intercalated with 1D BiOCl nanorods. BiOCl serves as a potential substitute for noble metals, accelerating the charge separation and transport of photo carriers. The resulting Bi_2_WO_6_/BiOCl composites showed superior visible light photodegradation on RhB and CIP, respectively. The quenching tests demonstrated that holes and ·OH play significant roles in the photocatalytic degradation process. The 3D hierarchical and hollow structure, together with the specific interfacial interaction, synergistically contributed to the enhanced photoactivity. This study provided a cost-effective approach to realize the critical and efficient separation of photoinduced carriers for environmental remediation applications.

## Supplementary Materials

The following are available online at https://www.mdpi.com/2079-4991/9/3/322/s1, Table S1: Kinetic parameters of RhB adsorption on BiOCl/Bi_2_WO_6_ hybrids, Table S2: Summary of nitrogen adsorption–desorption results of the prepared photocatalysts, Table S3: The valence band (VB) edge and the conduction band (CB) edge positions of BiOCl and Bi_2_WO_6_, Figure S1: Wavelength distribution of Xe lamps that were used in visible light photocatalytic reactions, Figure S2: FESEM images of the as-prepared samples: (a) 1.5Cl-0.75W, (b) 1Cl-1W, (c) 0.5Cl-1.25W, and (d) related XRD patterns, Figure S3: A schematic flow chart for the synthesis of BiOCl/Bi_2_WO_6_ hybrid materials. Figure S4: TEM analysis of 1.5Cl-0.75W composite, Figure S5: A representative series of UV-vis spectra of a series of photocatalytic tests with different concentration of RhB solution: (a) 2 M, (b) 3 M and (c) 5 M, respectively. Figure S6: Transient photocurrent density as a function of time in 0.5 M Na_2_SO_4_.
