# Supplementary material for: 3D Hollow Hierarchical Structures Based on 1D BiOCl Nanorods Intersected with 2D Bi2WO6 Nanosheets for Efficient Photocatalysis Under Visible Light
Source: Nanomaterials (Basel). 2019 Mar 1;9(3):322. doi: 10.3390/nano9030322 (PMC6473858; doi:10.3390/nano9030322)
Supplement: Supplementary file 1 [file nanomaterials-09-00322-s001.pdf]

# Supporting information

# 3D Hollow Hierarchical Structures Based on 1D BiOCl Nanorods Intersected with 2D $Bi_2WO_6$ Nanosheets for Efficient Photocatalysis Under Visible Light

**Yongchao Ma, Chao Lv, Jiahui Hou, Shaoteng Yuan, Yanru Wang, Ping Xu, Ge Gao and Jinsheng Shi ***

Department of Chemistry and Pharmaceutical Science, Qingdao Agricultural University, Qingdao 266109, China

* Correspondence: jsshiqn@aliyun.com; Tel.: +86-532-88030161; Fax: +86-532-86080213

**RhB dye adsorption**

RhB was employed for evaluating the adsorption capacity for $BiOCl/Bi_2WO_6$ hybrids. In brief, 20 mg of the hybrid was added into 100 mL of RhB solution (10 mg $L^{-1}$). Samples were collected at different time intervals and the RhB concentration ($C_t$) in the supernatant liquid was measured on a TU-1901 spectrophotometer. The amount of dye adsorbed ($q_t$) onto the hybrid was calculated as follows:

$$q_t = \frac{(C_0 - C_t)V}{m} \tag{1}$$

where $C_0$ is the initial concentration (mg $L^{-1}$) and $C_t$ is the concentration at time t (min), respectively. V is the volume of the solutions (L) and m is the weight of hybrids used (g) [1,2].

The linear forms of Lagergren-first-order and pseudo-second-order are represented by Equations (2) and (3), respectively.

$$\log(q_e - q_t) = \log q_e - \frac{k_1 - t}{2.303} \tag{2}$$

$$\frac{t}{q_t} = \frac{1}{k_2 q_e^2} + \frac{1}{q_e} t \tag{3}$$

where $q_e$ and $q_t$ are the amounts of RhB adsorbed (mg $g^{-1}$) at equilibrium and at time t (min), respectively; $k_1$ is the rate constant of Lagergren-first-order kinetic model ($min^{-1}$); $k_2$ is the rate constant (g $mg^{-1}$ $min^{-1}$) of pseudo-second-order kinetic model.

**Photocatalytic tests**

The photocatalytic activities of the as-prepared products were evaluated by the degradation of RhB and Ciprofloxacin (CIP) under visible light irradiation. In a typical experiment, 20 and 100 mg of photocatalysts were added to 100 mL of RhB aqueous solution with different concentrations ($10^{-5}$, $2 \times 10^{-5}$, $3 \times 10^{-5}$ and $5 \times 10^{-5}$ M) and CIP aqueous solution (100 mL, 10 mg $L^{-1}$), respectively. The photocatalytic reactor (PLS-SXE 300, Beijing Perfect light Co., Ltd., Beijing, China), consisting of a quartz glass with a circulating water jack and a 300W Xe lamp with a 420 nm cutoff filter. The light intensity striking the model pollutant solution was at ~23 mW $cm^{-2}$, as measured by a FZ-A optical Radiometer (Photoelectric Instrument Factory of Beijing Norman University, Beijing, China). The optical spectrum of the 300W Xe lamp with a 420 nm cutoff filter is given in Figure S1. Prior to irradiation, the suspensions were magnetically stirred in the dark for about 30 min to obtain a good adsorption–desorption equilibrium between photocatalysts and RhB (CIP). At certain time intervals, 5 mL of the solution was taken out and centrifuged to remove the photocatalysts. The concentrations of CIP and RhB were analyzed by recording variations of the characteristic absorption band of 272 and 553 nm using a TU-1901 spectrophotometer, respectively.

**Reactive species trapping experiments.**

It is well-known that in photocatalytic degradation reactions, hydroxyl (·OH) radicals, holes ($h^+$) and superoxide ($O_2{\cdot}^-$) radical anions are the major active species in the oxidation and reduction of target pollutants [3,4]. To determine the reactive radical species and holes involved in the degradation of RhB, trapping experiments were performed. The scavengers of ·OH, $h^+$ and $O_2{\cdot}^-$ species are isopropyl alcohol (IPA, 10 mM), triethanolamine (TEA, 6 mM) and 1,4-benzoquinone (BQ, 0.1 mM), respectively. The trapping experiment procedures were similar to that of the degradation experiment, except that the various scavengers were introduced separately to the RhB solution.

**Photoelectrochemical measurement**

Electrochemical impedance spectra (EIS) and photocurrent measurements were performed in an electrochemical work station (CHI660D). A standard three-electrode configuration was used with a saturated calomel electrode as the reference electrode and a Pt electrode as the counter electrode. 0.1 M $Na_2SO_4$ aqueous solution was utilized as the electrolyte. The light source used was the same as in photocatalytic tests. After the open circuit potential of the photoanode remained fairly constant over time, EIS measurements were conducted in the frequency range of $10^5$–$10^{-2}$ Hz at the open circuit potential with an alternating current voltage amplitude of 5 mV and a data density of twelve points per decade.

**Table S1.** Kinetic parameters of RhB adsorption on $BiOCl/Bi_2WO_6$ hybrids.

| **Kinetic model** | **$q_{e, exp}$ (mg g$^{-1}$)** | **$q_{e, cal}$ (mg g$^{-1}$)** | **k** | **$R^2$** |
|---|---|---|---|---|
| Langergren-first-order model | 27.92 | 26.71 | $k_1$ (min$^{-1}$) | 0.98975 |
| Pseudo-second-order kinetic model | 27.92 | 27.73 | $k_2$ (g mg$^{-1}$ min$^{-1}$) | 0.99718 |

**Table S2.** Summary of nitrogen adsorption–desorption results of the prepared photocatalysts.

| **Samples** | **BiOCl** | **$BiOCl/Bi_2WO_6$** | **$Bi_2WO_6$** |
|---|---|---|---|
| BET surface area ($m^2$ $g^{-1}$) | 41.74 | 43.14 | 95.06 |
| Average pore size (nm) | 10.84 | 11.47 | 7.630 |

**Table S3.** The valence band (VB) edge and the conduction band (CB) edge positions of BiOCl and $Bi_2WO_6$.

| | **X** | **$E^e$** | **$E_g$** | **$E_{VB}$** | **$E_{CB}$** |
|---|---|---|---|---|---|
| BiOCl | 6..34 | 4.5 | 3.22 | 3.45 | 0.23 |
| $Bi_2WO_6$ | 6.36 | 4.5 | 2.62 | 3.15 | 0.53 |

The valence band (VB) edge and the conduction band (CB) edge positions of BiOCl and $Bi_2WO_6$ can be calculated from the following formula:

$$E_{VB} = X - E^e + 0.5E_g \quad (4)$$

$$E_{CB} = E_{VB} - E_g \quad (5)$$

where $E_{VB}$ is the VB edge potential, $E_{CB}$ is the CB edge potential, $E_g$ is the band gap energy of the semiconductor, X is the electronegativity of the semiconductor that is the geometric mean of the electronegativity of the constituent atoms, $E^e$ is the energy of free electrons on the hydrogen scale (about 4.5 eV). The X values for BiOCl and $Bi_2WO_6$ were 6.34 and 6.36 eV, respectively [5–7]. Thus, the $E_{VB}$ and $E_{CB}$ of BiOCl were calculated to be 3.45 and 0.23 eV, respectively. The $E_{VB}$ and $E_{CB}$ of $Bi_2WO_6$ were calculated to be 3.15 and 0.53 eV, respectively.

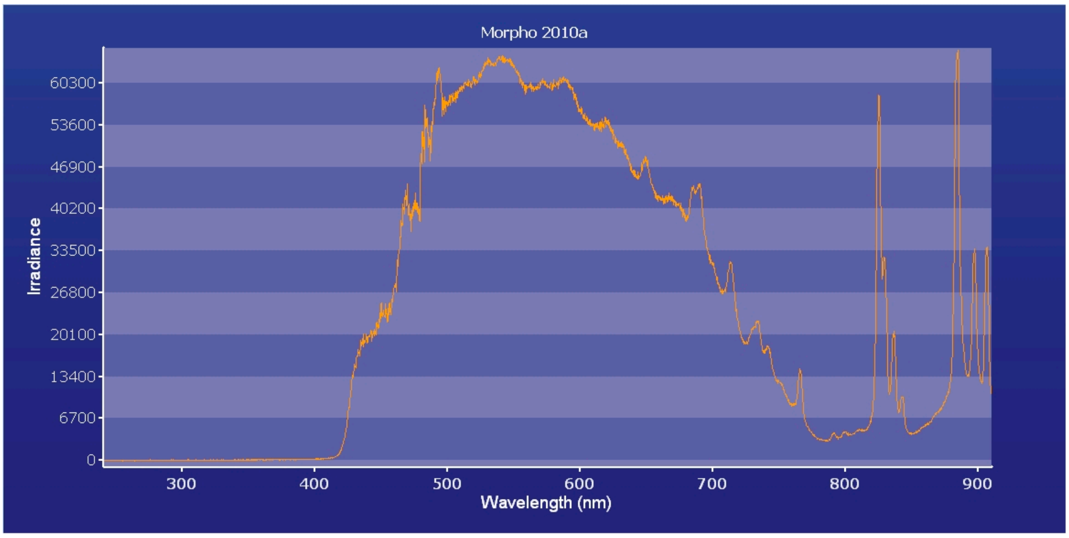


**Figure S1.** Wavelength distribution of Xe lamps that were used in visible light photocatalytic reactions.

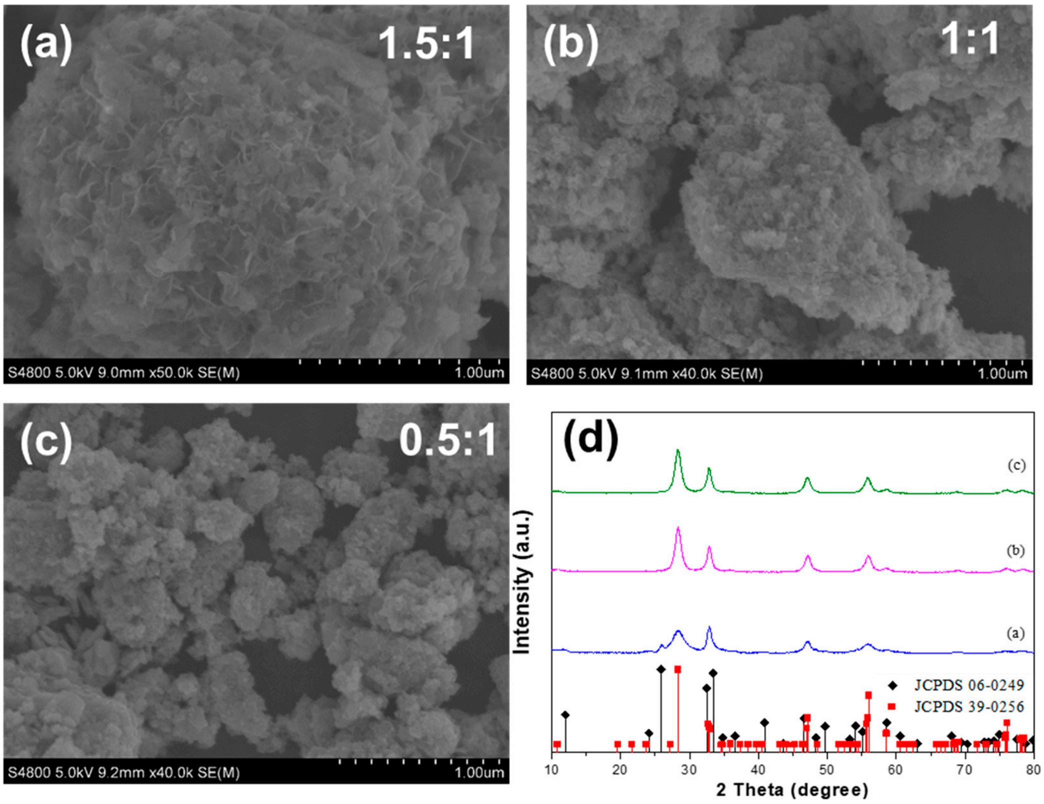


**Figure S2.** Field-emission scanning electron microscopy (FESEM) images of the as-prepared samples: (**a**) 1.5Cl-0.75W, (**b**) 1Cl-1W, (**c**) 0.5Cl-1.25W, and (**d**) related X-ray diffractometer (XRD) patterns.

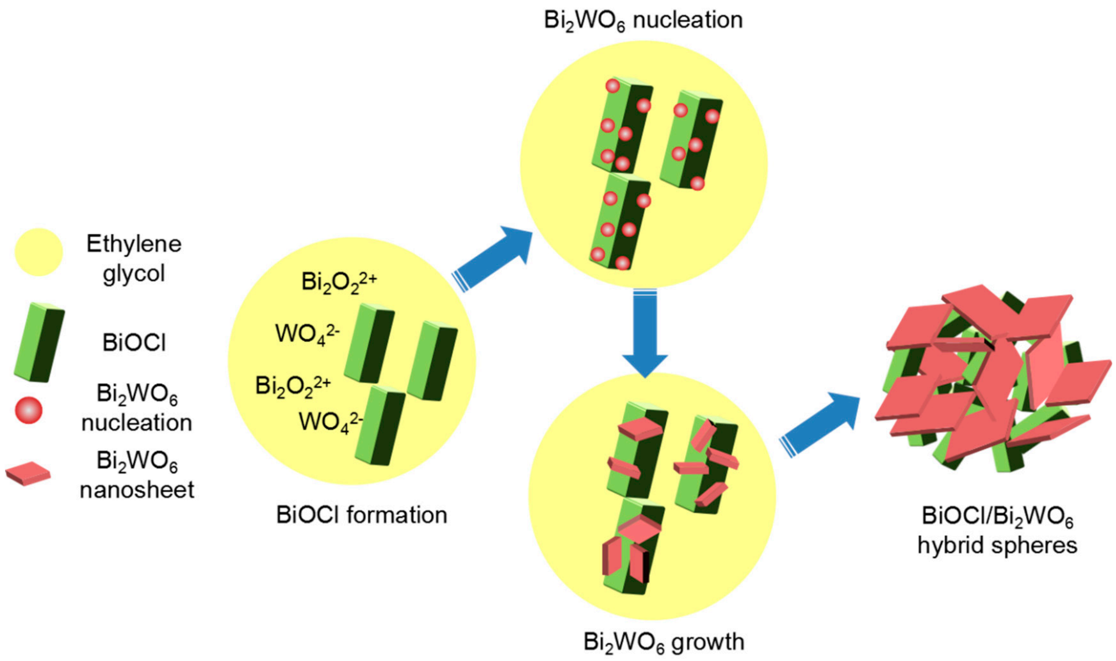


**Figure S3.** A schematic flow chart for the synthesis of BiOCl/$Bi_2WO_6$ hybrid materials.

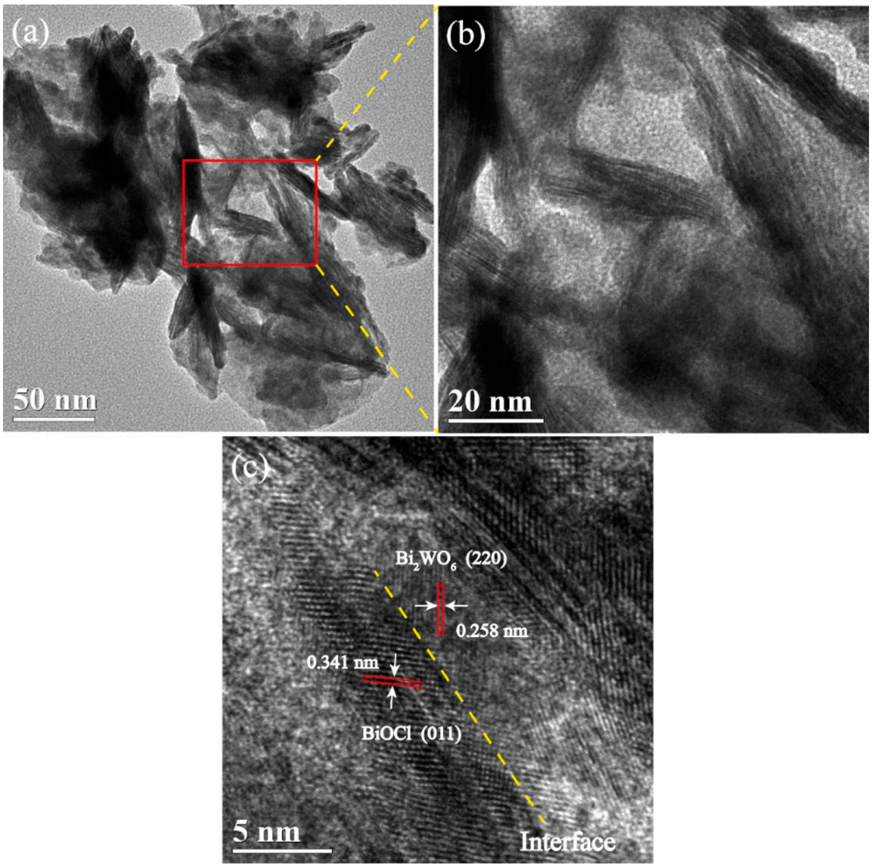


**Figure S4. T**ransmission electron microscopy (TEM) analysis of 1.5Cl-0.75W composite: (**a**) Low-magnification and (**b**) high-magnification transmission electron microscopy (TEM) images, (**c**) high-resolution transmission electron microscopy (HRTEM).

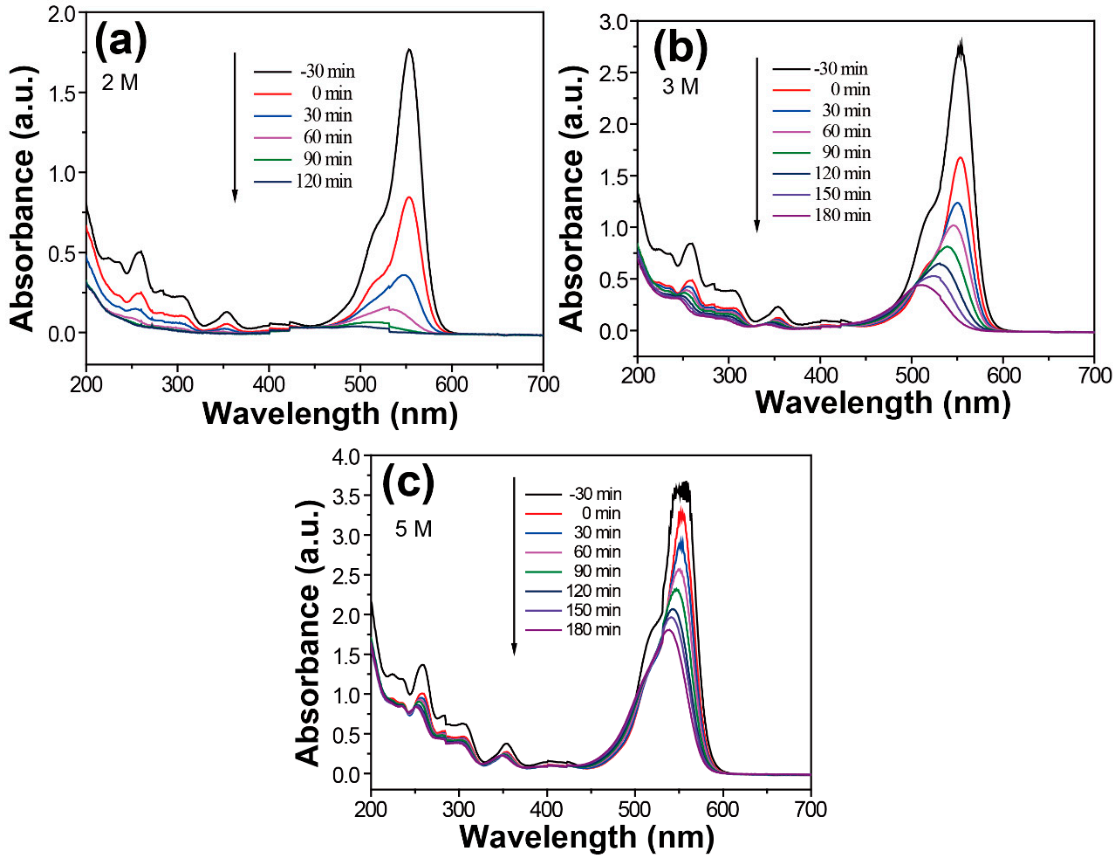


**Figure S5.** A representative series of UV-vis spectra of a series of photocatalytic tests with different concentration of RhB solution: (**a**) 2 M, (**b**) 3 M and (**c**) 5 M, respectively.

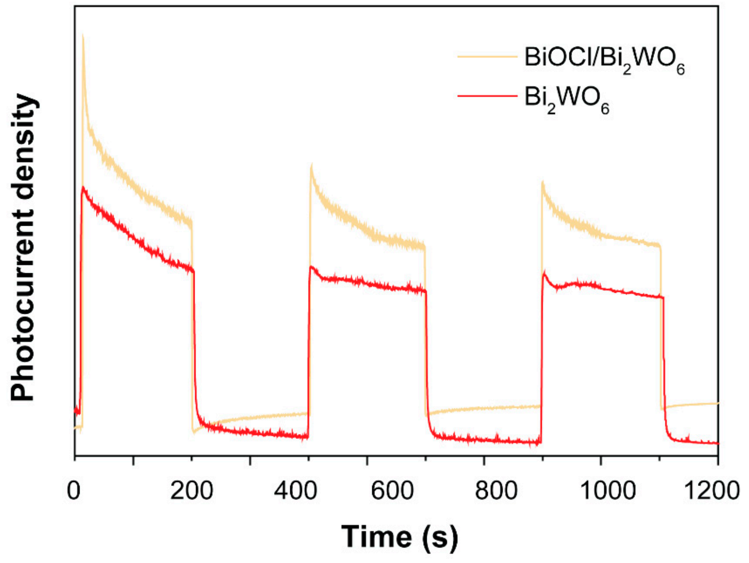


**Figure S6.** Transient photocurrent density as a function of time in 0.1 M $Na_2SO_4$.

## Supporting references

1. Zhu, H.; Jiang, R.; Xiao, L.; Liu, L.; Cao, C.; Zeng, G. CdS nanocrystals/$TiO_2$/crosslinked chitosan composite: Facile preparation, characterization and adsorption-photocatalytic properties. *Appl. Surf. Sci.* **2013**, *273*, 661–669.
2. Monte Blanco, S.P.D.; Scheufele, F.B.; Módenes, A.N.; Espinoza-Quiñones, F.R.; Marin, P.; Kroumov, A.D.; Borba, C.E. Kinetic, equilibrium and thermodynamic phenomenological modeling of reactive dye adsorption onto polymeric adsorbent. *Chem. Eng. J.* **2017**, 307, 466–475.
3. Jo, W.-K.; Sivakumar Natarajan, T. Facile synthesis of novel redox-mediator-free direct Z-scheme $CaIn_2S_4$ marigold-flower-like/$TiO_2$ photocatalysts with superior photocatalytic efficiency. *ACS Appl. Mater. Interfaces* **2015**, *7*, 17138–17154.
4. Chu, C.-Y.; Huang, M.H. Facet-dependent photocatalytic properties of $Cu_2O$ crystals probed by using electron, hole and radical scavengers. *J. Mater. Chem. A* **2017**, *5*, 15116–15123.
5. Chen, L.; Hua, H.; Yang, Q.; Hu, C. Visible-light photocatalytic activity of $Ag_2O$ coated $Bi_2WO_6$ hierarchical microspheres assembled by nanosheets. *Appl. Surf. Sci.* **2015**, *327*, 62–67.
6. Cao, J.; Xu, B.; Lin, H.; Luo, B.; Chen, S. Novel $Bi_2S_3$-sensitized BiOCl with highly visible light photocatalytic activity for the removal of rhodamine B. *Catal. Commun*. **2012**, *26*, 204–208.
7. Dong, F.; Sun, Y.; Fu, M.; Wu, Z.; Lee, S.C. Room temperature synthesis and highly enhanced visible light photocatalytic activity of porous BiOI/BiOCl composites nanoplates microflowers. *J. Hazard. Mater.* **2012**, *219–220*, 26–34.
